# Atrial Fibrillation

**DOI:** 10.1155/2013/142673

**Published:** 2013-03-03

**Authors:** Natig Gassanov, Evren Caglayan, Firat Duru, Fikret Er

**Affiliations:** ^1^Department of Internal Medicine III, University of Cologne, Kerpener Stra**β**e 62, 50937 Cologne, Germany; ^2^Clinic for Cardiology, University Hospital Zurich, 8091 Zürich, Switzerland

Atrial fibrillation (AF) is the most common clinically important cardiac arrhythmia. The prevalence of AF roughly doubles with each advancing decade of age, from 0.5% at age of 50–59 years to almost 9% at age of 80–89 years [1]. AF is associated with substantial morbidity and mortality. Thus, AF is a significant risk factor for ischemic stroke and accounts for 15–20% of all strokes [2]. Considering the clinical relevance of AF, this journal initiated a special issue dealing with the recent developments in AF over the past few years. This issue contains important work—both review and original articles—addressing the epidemiology, economic impact of AF, and new therapeutic/diagnostic developments and their potential clinical implications.

The general therapeutic strategies in AF include heart rate or rhythm control and anticoagulation. Current drugs used for AF therapy have major limitations, including incomplete efficacy and risks of life-threatening proarrhythmic events and bleeding complications. However, there have been several recent advancements in therapy of AF. They included the availability of new anticoagulants, such as dabigatran, rivaroxaban, and apixaban, as well as guideline changes to incorporate the catheter-based isolation of pulmonary veins (PV) as a class IIa/A indication [3]. Since the first paper evidencing the role of PV as triggers of AF [4], various ablation techniques targeting PV (focal ablation, PV isolation, circumferential antral ablation, cryoballoon) were introduced into clinical practice [5, 6]. Though PV isolation by catheter-based radiofrequency ablation has become an effective treatment option in AF, the studies on long-term outcomes are still limited and less encouraging.

Recently, F. Ouyang et al. examined 5-year outcomes in paroxysmal AF and found that sinus rhythm was present in 46.6% of patients after one procedure [7]. Long-term outcome data after catheter ablation for persistent AF are even less favourable [8]. These data highlight important aspects of catheter ablation, in particular the need for improved tools for patient selection, energy delivery for durable transmural lesions, and more studies on long-term outcomes and the management of very late recurrences as mediators of initial procedural approach refinement. Radiological investigations such as CT scan or cardiac magnetic resonance imaging (cMRI) can demonstrate the complex LA anatomy very well in all three dimensions and should reduce the fluoroscopy times. In this special issue, P. Haemers et al. review the potential application of cMRI in the diagnostic workup and treatment of AF. Besides its value in the guidance of catheter ablation, the authors describe the role of cMRI in identifying the underlying pathophysiological mechanisms of AF and in stroke prevention. cMRI can be used for left atrial scar quantification to assist not only in the procedural approach but also in patient selection.

As mentioned above, stroke is a major critical AF-associated complication. Today, stroke prevention with anticoagulant agents based on the CHADS_2_/CHA_2_DS_2_-VASc-Score is the main cornerstone of AF management. However, anticoagulation—even with newly developed oral anticoagulants—has several limitations. In addition to the bleeding risk, anticoagulation is not effectively utilized or contraindicated in significant number of eligible AF patients, often due to the limitations highlighted in the current paper by T. K. Patel et al. While newer oral anticoagulants overcome many of these limitations, all anticoagulants suffer from an unavoidable lifelong commitment to medication and elevated bleeding risk.

The left atrial appendage (LAA) is particularly vulnerable to thrombus formation due to its complex anatomy and low blood flow during AF. Therefore, LAA exclusion may be an especially appealing option for patients with intolerance or contraindications to anticoagulation. The procedure of LAA excision as well as the device characteristics are now highlighted in the current special issue. Indeed, LAA appendage may be a true alternative to anticoagulation in some patients. However, there is a very limited clinical experience with this novel procedure, and it is obvious that further studies are urgently required to clarify the benefits and disadvantages of LAA removal in patients with AF.

In another study of the current issue, C. J. Mercaldi et al. quantified the direct long-term costs, up to 3 years, of both stroke and bleeding events among patients with nonvalvular AF. Health care costs due to AF are enormous; on the basis of current US age- and sex-specific prevalence data, the national incremental AF cost is estimated to range from $6.0 to $26.0 billion [9]. The authors conducted a retrospective cohort study of Medicare beneficiaries newly diagnosed with AF who later developed stroke or hemorrhagic events. The results show that beyond the first year after the event, patients with major bleeding events other than intracranial hemorrhage have significantly higher costs than matched controls, underlying the need for proper cost-effectiveness assessment of the true long-term costs of stroke and major bleeding events. This strategy is particularly required when weighing the risks (bleeding) and benefits (stroke prevention) of anticoagulation in patients with AF.

In summary, we hope that expert contributions to this special issue will improve our understanding of the mechanisms of AF and ultimately result in a better clinical management of AF and long-term outcomes.

*Natig Gassanov* *Evren Caglayan* *Firat Duru* *Fikret Er*
